# Geographical clustering and geographically weighted regression analysis of home delivery and its determinants in developing regions of Ethiopia: a spatial analysis

**DOI:** 10.1186/s12982-022-00117-8

**Published:** 2022-08-19

**Authors:** Setognal Birara Aychiluhm, Mequannent Sharew Melaku, Kusse Urmale Mare, Abay Woday Tadesse, Getahun Fentaw Mulaw

**Affiliations:** 1 Department of Public Health, College of Medicine and Health Sciences, Samara University, Samara, Ethiopia; 2 Department of Health Informatics, Institute of Public Health, College of Medicine and Health Sciences, University of Gondar, Gondar, Ethiopia; 3 Department of Nursing, College of Medicine and Health Sciences, Samara University, Samara, Ethiopia; 4 School of Public Health, Woldia University, Woldia, Ethiopia

**Keywords:** Developing regions, Determinants, Ethiopia, Geographically weighted regression, Home delivery, Spatial analysis

## Abstract

**Background:**

Nearly three-fourths of pregnant women in Ethiopia give birth at home. However, the spatial pattern and spatial variables linked to home delivery in developing regions of Ethiopia have not yet been discovered. Thus, this study aimed to explore the geographical variation of home delivery and its determinants among women living in emerging (Afar, Somali, Gambella, and Benishangul-Gumuz) regions of Ethiopia, using geographically weighted regression analysis.

**Methods:**

Data were retrieved from the Demographic and Health Survey program's official database (http://dhsprogram.com). In this study, a sample of 441 reproductive-age women in Ethiopia's four emerging regions was used. Global and local statistical analyses and mapping were performed using ArcGIS version 10.6. A Bernoulli model was applied to analyze the purely spatial cluster discovery of home delivery. GWR version 4 was used to model spatial regression analysis.

**Results:**

The prevalence of home delivery in the emerging regions of Ethiopia was 76.9% (95% CI: 72.7%, 80.6%) and the spatial distribution of home delivery was clustered with global Moran’s I = 0.245. Getis-Ord analysis detected high-home birth practice among women in western parts of the Benishangul Gumz region, the Eastern part of the Gambela region, and the Southern and Central parts of the Afar region. Non-attendance of antenatal care, living in a male-headed household, perception of distance to a health facility as a big problem, residing in a rural area, and having a husband with no education significantly influenced home delivery in geographically weighted regression analysis.

**Conclusions:**

More than three-fourths of mothers in the developing regions of Ethiopia gave birth at home, where high-risk locations have been identified and the spatial distribution has been clustered. Thus, strengthening programs targeted to improve antenatal care service utilization and women’s empowerment is important in reducing home birth practice in the study area. Besides, supporting the existing health extension programs on community-based health education through home-to-home visits is also crucial in reaching women residing in rural settings.

## Background

The majority of maternal deaths take place in developing nations, making maternal mortality a global health concern [1, 2]. Globally, more than 10.7 million women have died due to birth-related causes. Of these, 99% occurred in emerging regions and 66% were contributed by Sub-Saharan Africa [3]. In Ethiopia, 11,000 mothers died due to pregnancy and birth complications [4], although more than 80% of deaths are from preventable causes [5].

Studies revealed that delivery by skilled birth attendants reduces maternal mortality by more than three-fourths [6–8] but the majority of women in developing countries give birth at home [6, 9, 10]. For instance, more than half of deliveries in Sub-Saharan Africa took place at home [4, 11, 12]. In Ethiopia, the prevalence of home delivery ranges between 25.3 and 83.3% [13–16]. Moreover, the magnitude of home delivery in the four emerging regions of the country is unacceptably higher compared to other regions. For instance, 85% and 82% of mothers in the Afar region and Somali region respectively gave birth at home [13, 17].

Previous studies have identified factors associated with home delivery. These include; maternal age, maternal education level, respondent working status, husband education level, wealth index, residence site, number of antenatal care (ANC) visits, distance to a health facility, parity, and exposure to mass media [17–22].

Ethiopia has implemented a variety of initiatives and interventions to promote maternal health, including the development and deployment of health care personnel, community mobilization activities through the Health Extension Program, and the growth of primary healthcare facilities [23], Millennium Development Goals [24], Health Sector Transformation Plan [25], and Sustainable Development Goals [26]. However, still, more than three-fourths of women give birth at home, particularly this problem is underreported and estimated in the emerging regions of the country.

Previous studies on the factors affecting home birth practice in the emerging regions of Ethiopia were equivocal and restricted to specific geographic locations [16, 27–33]. Besides, the spatial pattern and spatial covariates associated with home delivery in these regions were not identified so far. Identifying high-risk geographic areas and understanding the causes of disparities for high home birth practice by conducting a spatial and geographic weighted regression analysis is an important footstep in designing context-specific evidence-based interventions. Therefore, this study aimed to explore the geographical variation of home delivery and its determinants among women living in emerging (Afar, Somali, Gambella, and Benishangul-Gumuz) regions of Ethiopia, using geographically weighted regression analysis.

## Methods

### Study area and data source

The study was conducted in emerging regions (Afar, Somali, Gambella, and Benishangul-Gumuz regions) of Ethiopia. These regions are found mainly in lowland parts of the country and their main lifestyle depends on animal livestock and farming. The societies that exist in these areas are nomadic ethnic groups and highly moveable which are not suitable for the existing health system of the country [27, 34, 35]. As a result, these regions were not well realizing most of the health and development-related indicators compared to other developed regions of the country [36]. Besides, in these regions, maternal health care (antenatal care, skilled delivery care, postnatal care, and contraceptive) utilizations are influenced by socio-cultural and religious barriers [27, 30, 37, 38].

The data for this analysis were retrieved from the Demographic and Health Survey (DHS) program's official database website (http://dhsprogram.com), which was collected from January 18, 2016, to June 27, 2016. A total of 441(weighted sample) women living in four emerging (Afar, Somali, Gambella, and Benishangul-Gumuz) regions of Ethiopia who had at least one live birth in the 5 years preceding the survey were included in this analysis [13].

### Study variables

The outcome variable for this study was home delivery which was dichotomized into “Yes = 1 (for women whose last childbirth occurred at home) and No = 0 (for women whose last childbirth took place at health facilities)”. The independent variables were the sex of household head, age of respondent, marital status, birth order, women’s education level, husband’s education level, wealth index, respondent’s occupation, husband’s occupation, religion, exposure to mass media, antenatal care visit, type of residence, and distance to the health facility.

### Data management and statistical analysis

Sample allocation in the Ethiopian Demographic and Health Survey (EDHS) to different regions of the country as well as urban and rural areas was not proportional. Thus, this study applied sample weights to estimate proportions and frequencies to adjust disproportionate sampling and non-response. A full clarification of the weighting procedure was explained in the 2016 EDHS report [13]. The data cleaning was executed using Stata version 16.0 and MS-excel 2019.

### Spatial analysis

The spatial autocorrelation (Global Moran’s I) statistic was held to assess the pattern of home delivery whether it was dispersed, clustered, or randomly distributed in the study areas. Local Moran’s I measure positively correlated (High-High and Low-Low) clusters and outliers (High-Low: a higher value is surrounded primarily by lower values, and Low–High: a lower value is surrounded primarily by higher values). The detail about its statistical determination of cluster outlier is found in this literature [39, 40].

### Hot spot and cold spot analysis (Getis-Ord Gi* statistics)

Gettis-Ord Gi* statistics were calculated to measure how spatial autocorrelation differs through the study location by computing Gi* statistics for each area. Z-score was calculated to ensure the statistical significance of clustering and the p-value was calculated. To determine the statistical significance of clustering, Gi Z-score was calculated. A positive z-score > 1.96 with significant p-values denotes hot-spot, while negative Z-score <  − 1.96 with significant p-values denotes cold-spot [41, 42].

### Spatial regression analysis

Spatial regression was done using both local and global analysis techniques [43–45]. Therefore, a first global geographical regression model was applied, and then a local geographical analysis to ensure the variability of coefficients across each cluster [46–48]. Then, the six assumptions recommended for spatial regression were checked with the respective tests [49, 50]. Koenker Bp test was also executed to check whether the model underwent fitted geographically weighted regression (GWR) or not. GWR was executed using GWR version 4. Variables with a p-value less than 0.05 were selected as the determinants of home delivery and described based on their coefficients.

### Ethical consideration

The data access was obtained from the Demographic and Health Survey (DHS) website (http://www.measuredhs.com) after getting registered and permission was got. The retrieved data were used for this registered research only. The data were treated as confidential and no determination was made to identify any household or individual respondent.

## Results

### Socio-demographic and obstetrics characteristics

Out of the total respondents, 370 (83.9%) women were living in rural settings, 337 (76.5%) did not attend formal education, and 322 (72.9%) were from households with a poor wealth index. In this study, 356 (80.8%) respondents did not have exposure to mass media, and the decisions on health care for 212 (51.0%) women were jointly made with their husbands (Table 1).

**Table 1:** Socio-demographic, economic, and obstetrics characteristics of women in emerging regions of Ethiopia, 2016

Variable	Category	Frequency	Percent
Sex of the household head	Male Female	303 138	68.6 31.4
Marital status	Married Others +	415 26	94.0 6.0
Residence	Urban Rural	71 370	16.1 83.9
Religion	Muslim Others + +	373 68	84.6 15.4
Distance to a health facility	Big problem Not big problem	238 203	54.0 46.0
Respondent’s educational status	No education Primary Secondary and above	337 78 26	76.5 17.7 5.8
Husband’s educational status	No education Primary Secondary and above	280 79 58	67.1 18.9 14.0
Husband’s occupation	Have no work Have work	115 302	27.5 72.5
Respondent’s Currently working	No Yes	339 102	76.9 23.1
Wealth Index	Poor Middle Rich	322 35 84	72.9 8.0 19.1
Media Exposure	No Yes	356 85	80.8 19.2
Birth order	1–6 Above 6	340 101	77.1 22.9
Decision on health care	Women Jointly Husband	101 212 103	24.1 51.0 24.9

Other +  = Never in a union, Widowed, and Divorced; Others +  +  = Orthodox, Protestant, Catholic, Traditional, and other EDHS categories

### Spatial variation of home delivery in developing regions of Ethiopia

**Fig. 1:**
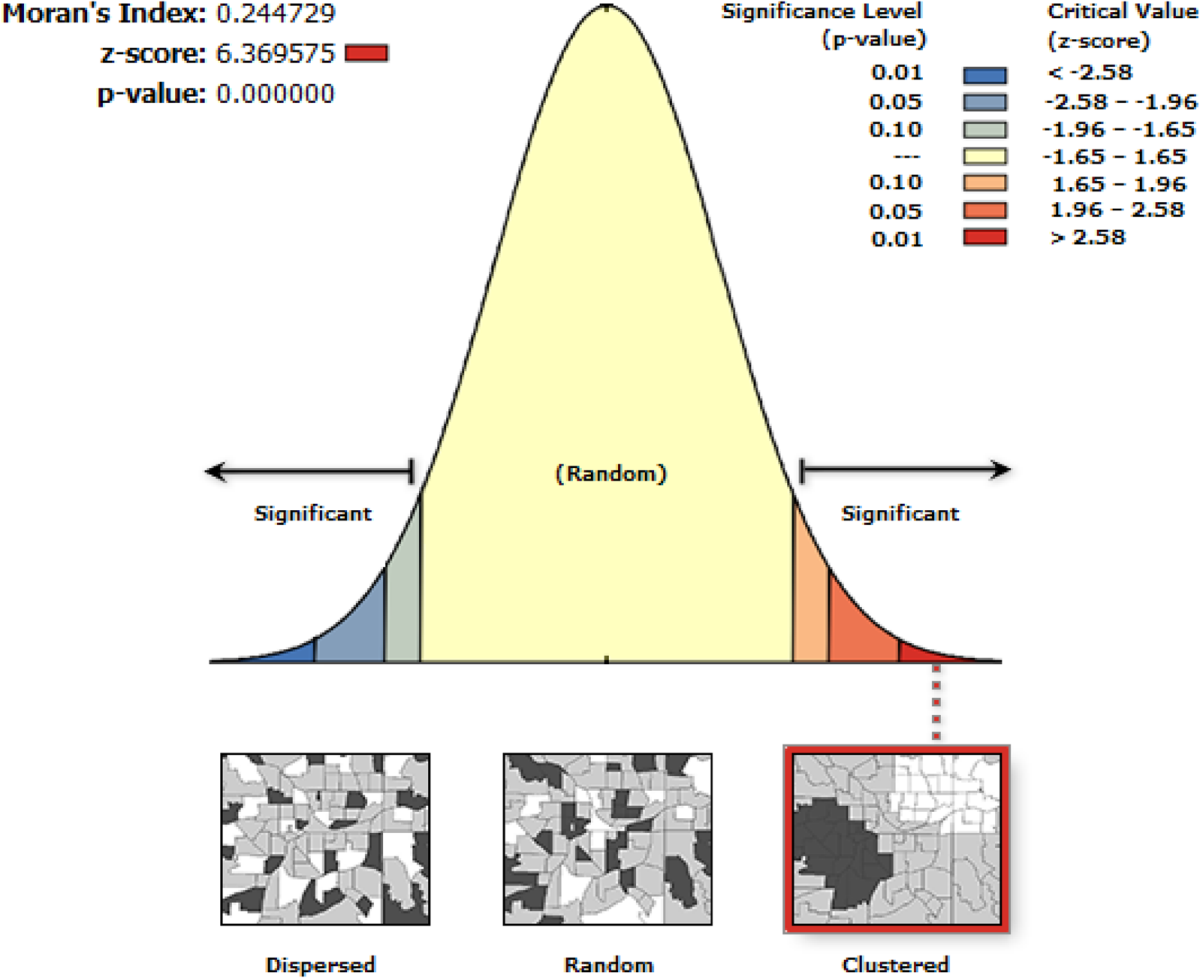
Spatial autocorrelation of home delivery among women in emerging regions of Ethiopia, 2016

The spatial distribution of home delivery was clustered at the cluster level in emerging regions of the country. Hence, the global Morans I index value was 0.245 (p-value < 0.001) for home delivery (Fig. 1).

### Spatial case distributions of home delivery in developing regions of Ethiopia

**Fig. 2:**
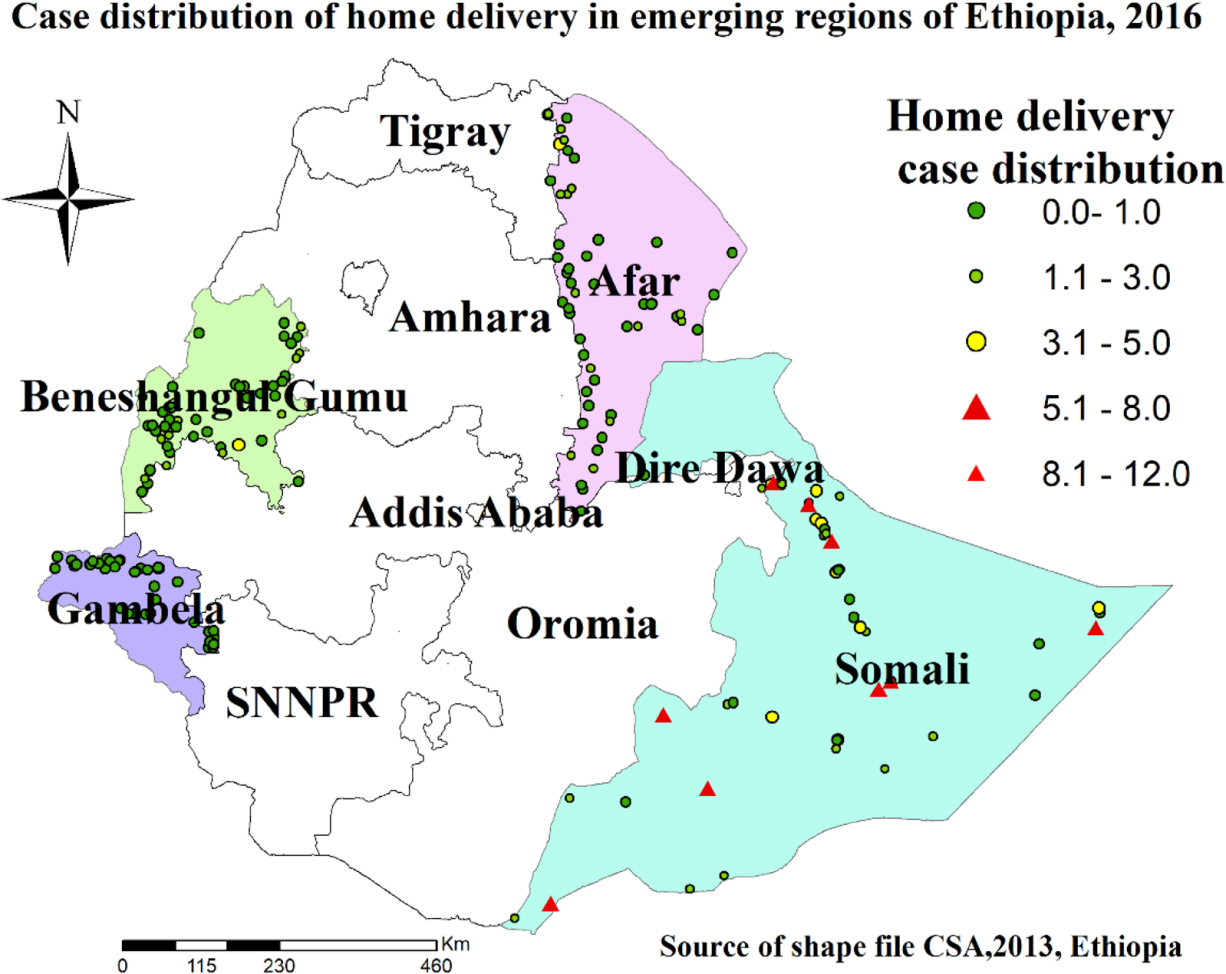
Spatial case distributions of home delivery among women in emerging regions of Ethiopia

Figure 2 showed that spatial variation in home birth practice was found at regional levels. For instance, the red dots indicate areas with a higher proportion of home delivery and the green dots show areas with a lower proportion of home delivery.

### Cluster and outlier cluster detection for home delivery

**Fig. 3:**
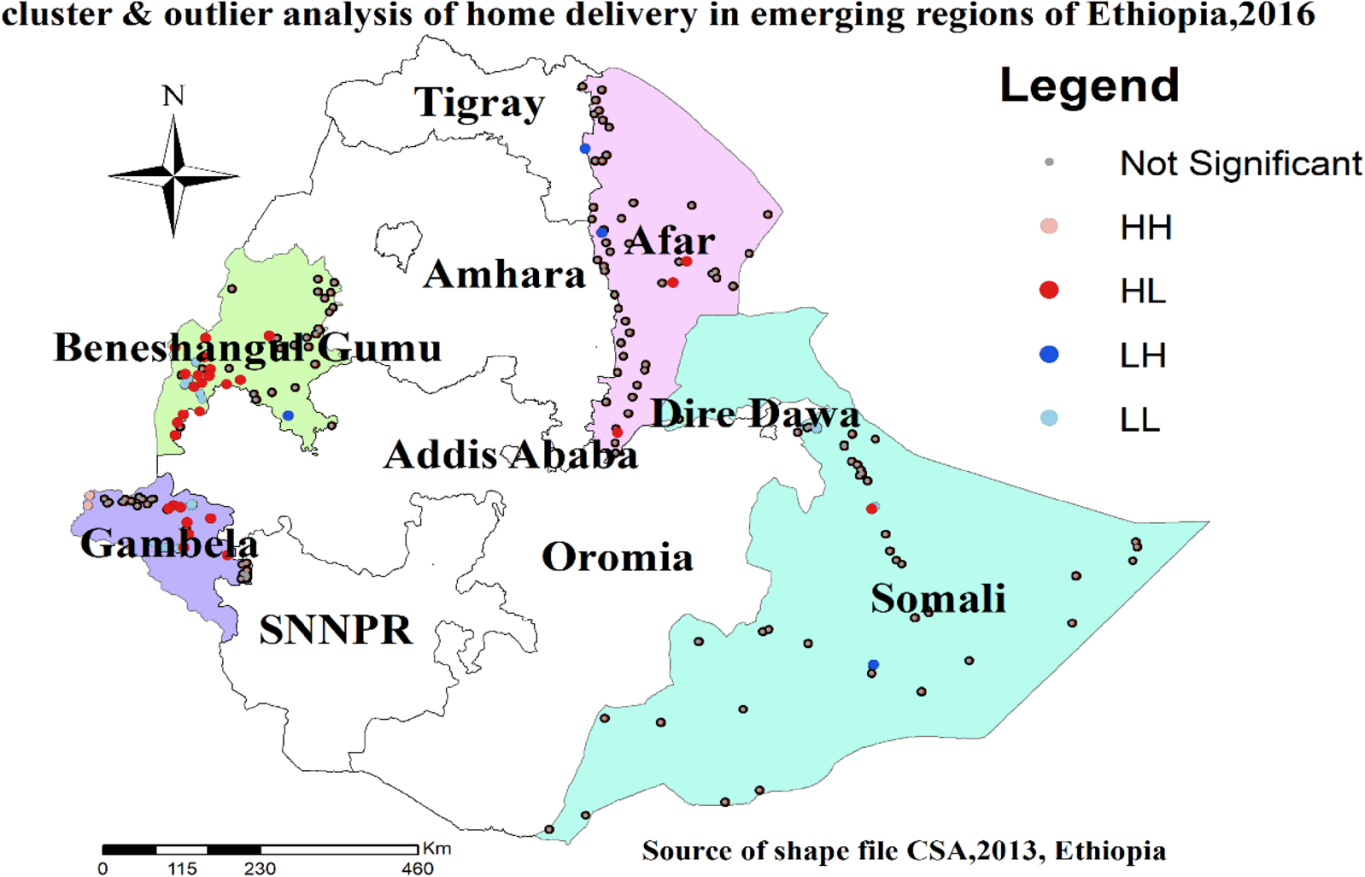
Cluster and outlier analysis of home delivery among women in emerging regions of Ethiopia

Local Moran’s I analysis result of home delivery revealed that there were significant outliers. Accordingly, high outliers for home delivery were detected in western parts of Benishangul Gumz, the Eastern part of Gambela, and the Southern and Central parts of the Afar regions. Western parts of Afar, southern Benishangul Gumz, and the central Somali region were detected as a low outlier for home delivery (Fig. 3).

### Hot spot and cold spot clusters for home delivery in developing regions of Ethiopia

**Fig. 4:**
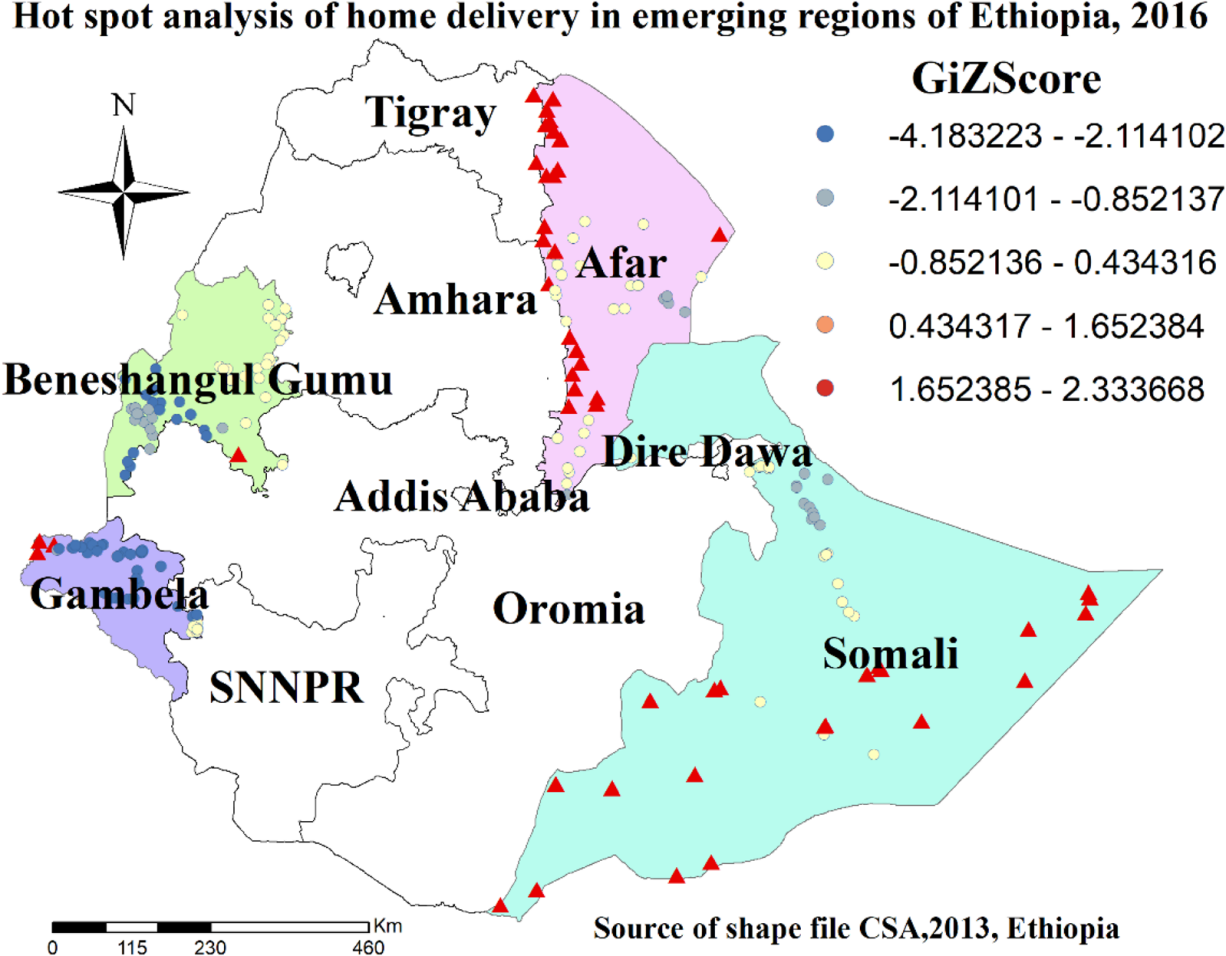
Hot spot and cold spot clusters of home delivery in emerging regions of Ethiopia, 2016

Hot spot analysis enables the detection of clusters with extreme high and low home delivery practices. Accordingly, hot spot (high risk) areas for home delivery were detected in the Afar region (Western border), Somali region (Western, Southwestern, Southern, and Eastern parts), Gambela region (Northwestern part), and some Southern parts of Benishagul Gumz region. On the other hand, the Gambela region (Northern, Central, and Eastern parts), and the Benishangul Gumz region (Southwestern and Southern parts) were detected as cold spot areas for home delivery (Fig. 4).

### Determinants of spatial variations of home delivery (Spatial regression analysis)

#### Ordinary least square

After checking spatial regression assumptions for home delivery using exploratory regression, an ordinary least square analysis was carried out. Outputs from the spatial regression analysis revealed that residuals of spatial relationships are uncorrelated and there was no multi-collinearity among explanatory variables. In ordinary least square analysis, women who did not have ANC visit, women whose husband was uneducated, perception of distance to a health facility as a big problem, residing in rural areas, and living in a male-headed household increased home delivery by 0.401, 0.182, 0.107, 0.196, and 0.107 times, respectively (Tables 2, 3).

**Table 2:** Spatial regression summary result of ordinary least square (global GWR) coefficients for home delivery in emerging regions of Ethiopia, 2016

Variables	Coefficient	Standard error	Probability	Robust probability	VIF
Intercept	− 15.67	5.59	0.06	0.0.09	–
Male household head	0.107	0.051	0.000	0.017	1.148
No ANC Visit	0.401	0.055	0.000	0.000	3.313
No husband education	0.182	0.046	0.000	0.000	1.878
Rural residence	0.196	0.032	0.000	0.000	1.712
Distance to a health facility is a big problem	0.107	0.045	0.019	0.036	1.763

**Table 3:** Spatial regression summary result of ordinary least square (global GWR) diagnostics for home delivery in emerging regions of Ethiopia, 2016

Diagnostics criteria	Magnitude	p-value
AICc	1811.6	
R squared	0.766	
Adjusted R squared	0.758	
Joint f statistics	99.1	0.0001*
Joint wald statistics	877.5	0.0001*
Koenker (Bp) statistics	22.8	0.001*
Jareque-Bera statistics	19.4	0.0925

### Geographically weighted regression of home delivery

**Fig. 5:**
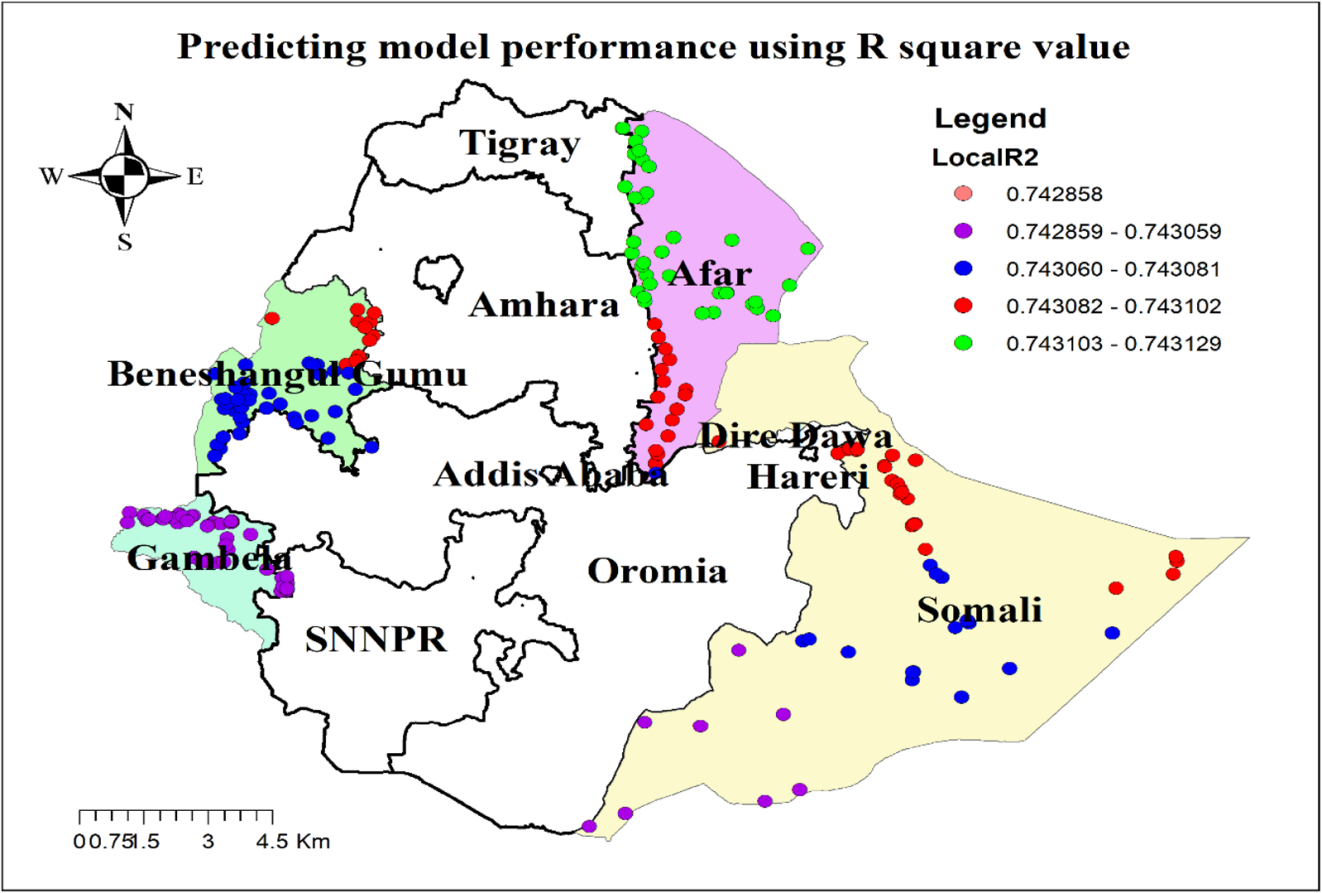
R square for showing model performance on predicting home delivery in emerging regions of Ethiopia, 2016

**Fig. 6:**
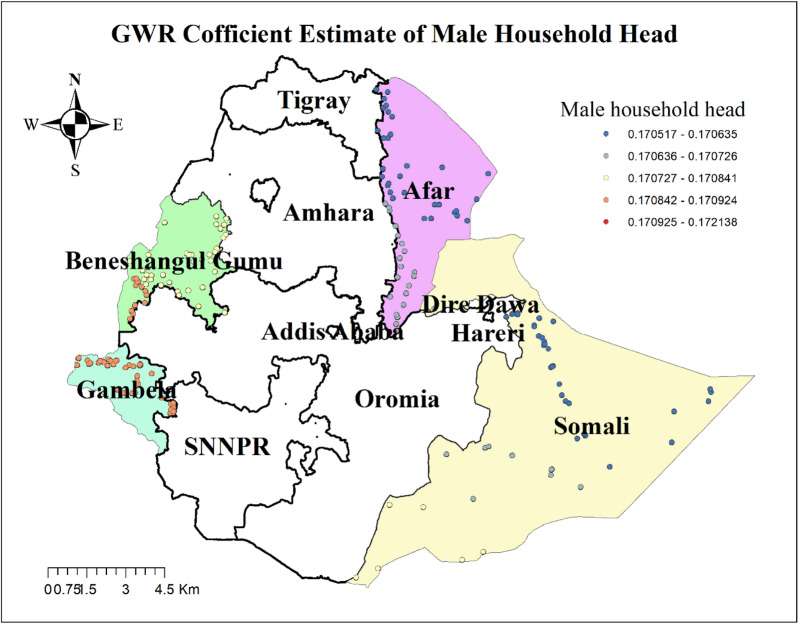
GWR coefficients for male-headed households on predicting home delivery in emerging regions of Ethiopia, 2016

**Fig. 7:**
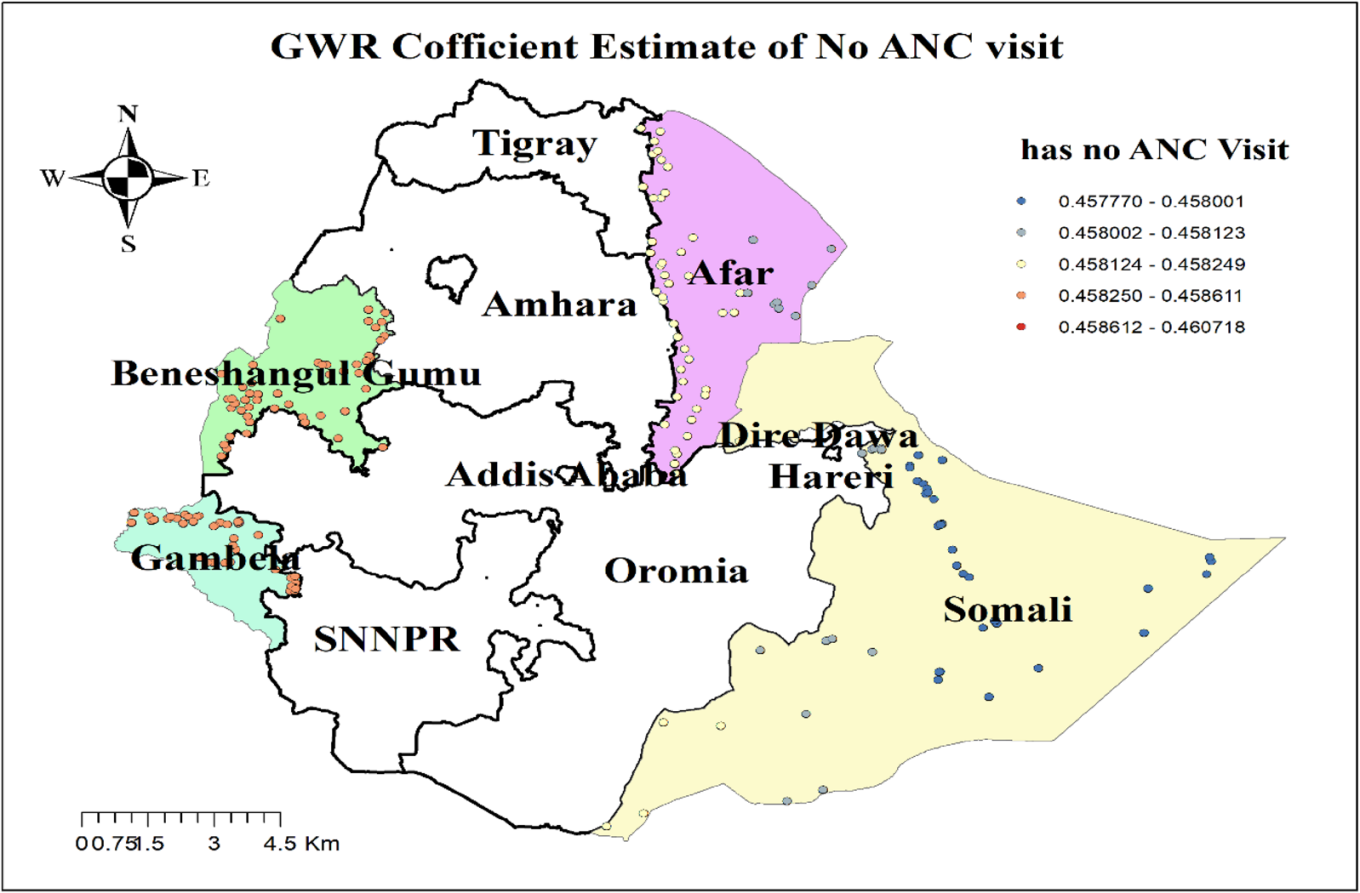
GWR coefficients for non-attendance of antenatal care visits on predicting home delivery in emerging regions of Ethiopia, 2016

**Fig. 8:**
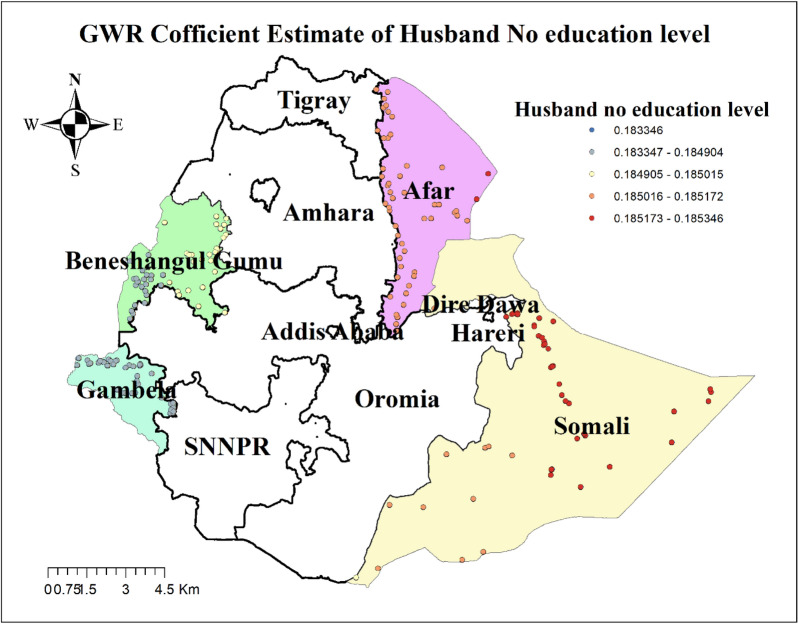
GWR coefficients for husbands with no education on predicting home delivery in emerging regions of Ethiopia, 2016

**Fig. 9:**
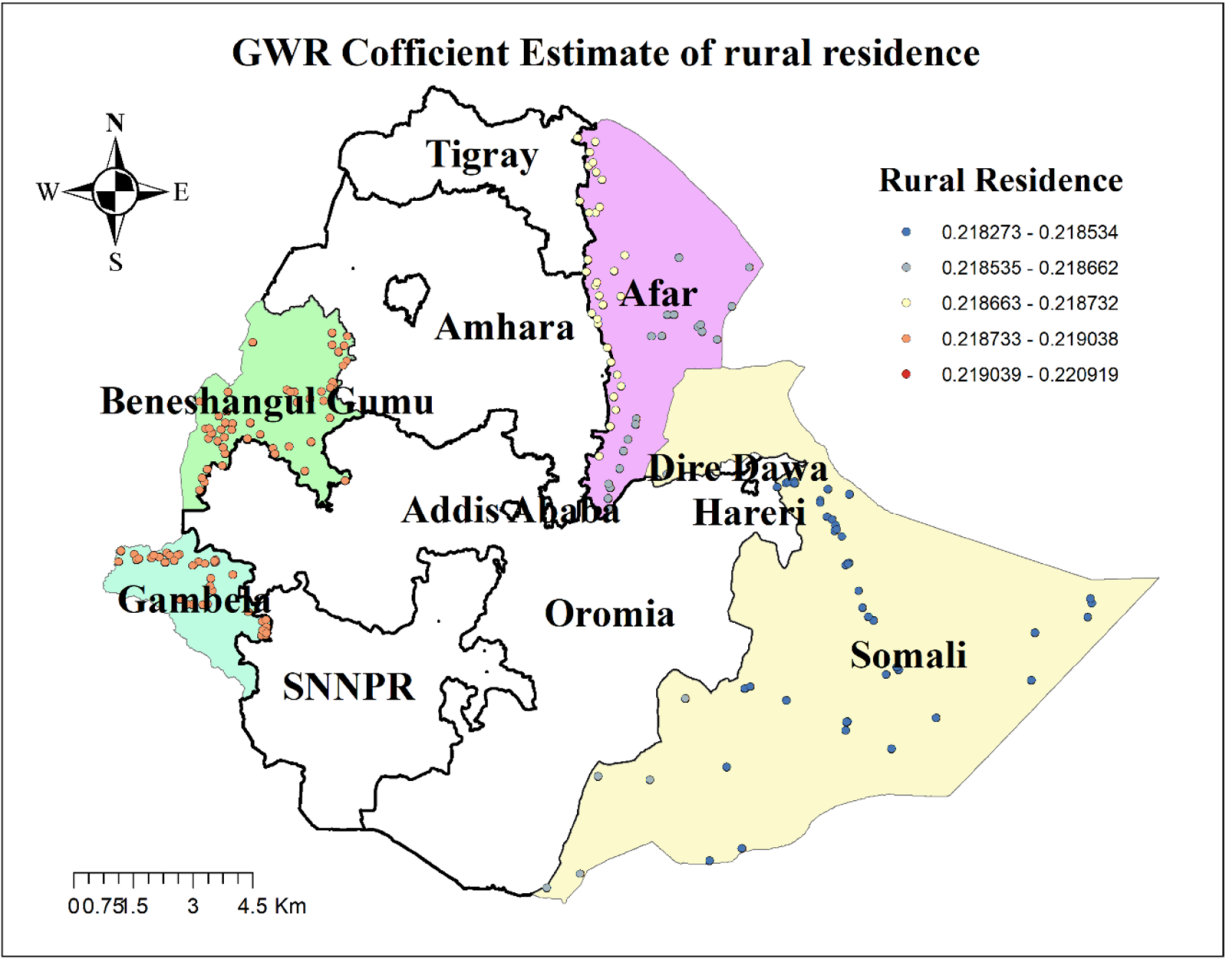
GWR coefficients for rural residence on predicting home delivery in emerging regions of Ethiopia, 2016

**Fig. 10:**
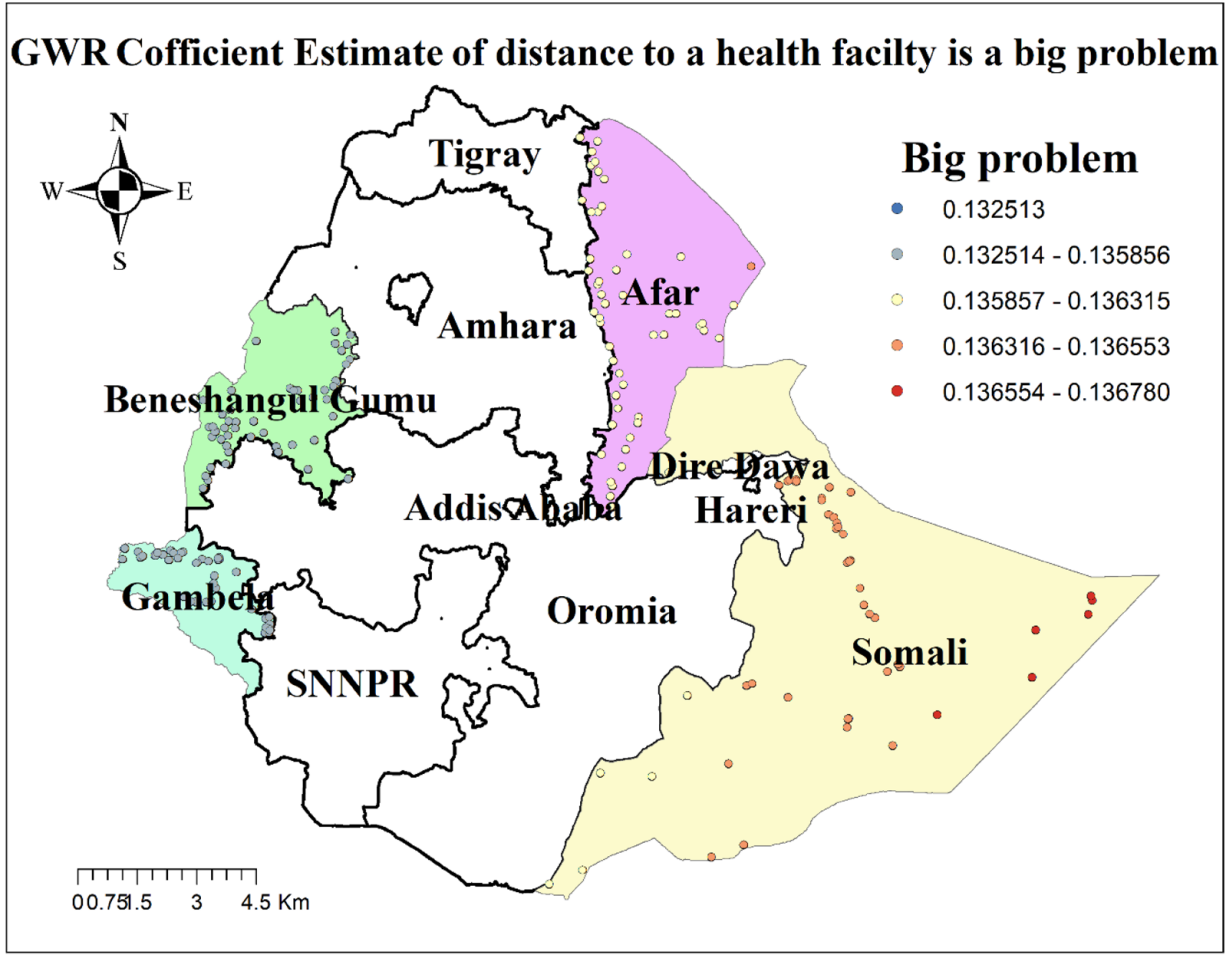
GWR coefficients for the perception of distance to a health facility a big problem in predicting home delivery in emerging regions of Ethiopia, 2016

The result of geographically weighted regression analysis identified different variable coefficients for the variables found in the ordinary least square analysis. Higher coefficients of having no ANC visit and residing in rural settings were detected in all parts of the Benshangul Gumz and Gambella regions. Similarly, higher coefficients for women who declared distance as a big problem were detected in all parts Somali region. Higher coefficients for women with uneducated husbands were detected in Afar and Somali regions. Higher coefficients for a household headed by a male were detected in Southern Benshangul Gumz and all parts of the Gambella region (Figs. 5, 6, 7, 8, 9, 10).

## Discussion

In Ethiopia, maternal mortality is not declined in the needed manner because of low maternal health service utilization, in which the causal mechanism has been intervening at the national level [51, 52]. In this study, more than three-fourths (76.9%, 95% CI: 72.7%–80.6%) of women gave birth at home. This finding is in line with other studies conducted in Arba Minch, Ethiopia (79.4%) [53], Benishangul Gumz, Western Ethiopia (80%) [15], Afar region, Ethiopia (83.3%) [16], Gozamin district, Northwest Ethiopia (75.3%) [54], Hadiya Zone, Southern Ethiopia (73.6%) [18], EDHS-2016 report (73%) [13], and Nigeria (74.1%) [55].

However, this finding is higher than studies done in Southern Tigray, Ethiopia (28.8%) [56], review study (67.2%) [17], Central Ethiopia (38.4%) [57], mini EDHS-2019 (52%) [58], Bench Maji Zone, Southwest Ethiopia (61.9%) [59], Debremarkos Town, Northwest Ethiopia (25.3%) [14], Bale zone, Southeast Ethiopia (67.1%) [60], and Zala district, Southern Ethiopia (67.6%) [61]. This discrepancy might be because the current study is carried out in the emerging regions of the country, where women have limited access to health care services due to their nomadic livelihood. Besides, in these regions, women’s exposure to the health information on maternal health care services is greatly affected by limited health infrastructure, transportation problems, and behavioral, socio-cultural, and religious preferences [55]. Thus, the prevalence of home delivery is found to be higher in these regions compared to the relatively developed regions in the country.

This study also showed that home delivery was clustered spatially at the enumeration area level. Getis-Ord spatial analysis showed that hot spot, cold spot, and outlier enumeration areas were detected using cluster outlier analysis. Different studies also prevailed on the existence of geographical clustering for home delivery [62–64]. The most possible explanation for this spatial variation could be the geographical variation of the country which ranges from 4550 m above sea level to 110 m below sea level. Consequently, infrastructure differences like road, electricity, water, the distribution of health facilities, and health care professionals across regions might have contributed to this variation. Besides, there is a difference in program implementation and distribution, socio-demographic characteristics, culture, community knowledge, and attitude towards home birth practice across different regions. Overall, these differences might have resulted in geographic inequalities of home delivery across different regions of the country.

In spatial regression analysis, different factors were found to have a statistically significant effect on home delivery. Accordingly, not attending ANC visits, being from male-headed households, perceiving distance to a health facility as a big problem, living in a rural residence, and having a husband with no education were the significant predictors for home delivery. Home birth practice was positively correlated with the non-attendance of antenatal care visits. Geographical areas identified for higher coefficients of women with no ANC visit were fitted with hot spots areas of home delivery. This might be because women who did not receive antenatal care miss the opportunity to get health information on the consequence of home delivery and the advantages of skilled delivery care that could result in their preference of home delivery.

Husband illiteracy was also positively correlated with home delivery. Geographical areas identified for higher coefficients of women whose husbands did not attend formal education were fitted with hot spots areas of home delivery. This finding is supported by a study conducted in developing countries that reported the positive effect of a partner’s education on maternal health care utilization [65]. This might be because educated husbands might have a better awareness of the benefits of maternal health care services which enables them to encourage their spouse to use these services. Thus, compared to women with educated husbands, women with uneducated husbands had an increased likelihood of giving birth at home.

Increased occurrence of home deliveries was also observed among women who were from a male-headed household. This finding can be explained by the fact that women in a male-headed household might have limited participation in household decisions due to gender-based power and economic inequalities which in turn influence their healthcare-seeking behavior [66, 67].

Moreover, living in a rural residence was positively correlated with home delivery. This might be because due to inequity in geographic access to healthcare services across different residences [68]. Thus, women residing in rural settings might face challenges in accessing health facilities which negatively influences their opportunity to get appropriate health information on maternal healthcare services. Likewise, in the context of distance, the possible reasons for home deliveries among rural women might be due to the mothers’ perception, sudden onset of labor, and inaccessible transportation [69].

### Strengths and limitations of the study

The analysis of this study was based on nationally representative and most recent EDHS data, which was collected by standardized and validated data collection instruments. Besides, the use of Geographic Information System (GIS) and Sat Scan statistical tests helped to detect similar and statistically significant high-risk clusters of home delivery. Moreover, the use of geographic weighted regression analysis helps to show the real impact of predictors in each specific geographic area. As a limitation, it was challenging to pinpoint the actual location of the cases since the location data values were shifted by 1–2 km for urban and 10 km for rural areas due to data confidentiality concerns.

### Implications of the study

This study adds to the existing body of information about how regional characteristics affect home delivery in Ethiopia's developing regions. This study helps to pinpoint specific hotspot locations throughout Ethiopia's developing regions and variables that have a big impact on home delivery, which is crucial for intervention. Even while there is evidence that elements like infrastructure coverage and geographic features have an impact, it can be challenging to identify the specific hotspot locations for home delivery where this study may have a solution.

## Conclusions

In the emerging regions of Ethiopia, more than three-fourths of women gave birth at home. This study showed that the distribution of home delivery was clustered at the enumeration area level in the emerging region of Ethiopia. Accordingly, hot spot (high-risk) regions for home delivery were detected in the Afar region (western border), Somali region (Western, Southwestern, Southern, and Eastern parts), the Gambella region (Northwestern part), and some Southern parts of Benishagul Gumz region.

Spatial regression analysis revealed that non-attendance of antenatal care visits, being from male-headed households, perceiving distance to a health facility as a big problem, residing in a rural setting, and having an uneducated husband were the significant determinants of home delivery in the emerging regions of Ethiopia. Thus, strengthening programs targeted to improve antenatal care service utilization and women’s empowerment is important in reducing home birth practice in the study area. Besides, supporting the existing health extension programs on community-based health education through home-to-home visits is also crucial in minimizing the distance barrier and reaching women residing in rural settings.

## Data Availability

The dataset used and analyzed in this study is available from the DHS program official database (http://dhsprogram.com).

## Abbreviations

ANC: Antenatal Care
DHS: Demographic and Health Survey
EDHS: Ethiopian Demographic and Health Survey
GWR: Geographically Weighted Regression
